# NCS-1 protein regulates TRPA1 channel through the PI3K pathway in breast cancer and neuronal cells

**DOI:** 10.1007/s13105-024-01016-z

**Published:** 2024-04-02

**Authors:** Julio C. Sánchez, Alexander Alemán, Juan F. Henao, Juan C. Olaya, Barbara E. Ehrlich

**Affiliations:** 1 Department of Basic Sciences, Laboratory of Cell Physiology, Faculty of Health Sciences, Universidad Tecnológica de Pereira, AA 97, La Julita, 660003 Pereira, Risaralda Colombia; 2 Departments of Pharmacology and Cellular and Molecular Physiology, Yale University, New Haven, CT 06520 USA

**Keywords:** Neuronal calcium sensor 1, NCS-1, Transient receptor potential channel ankyrin 1, TRPA1, Electrophysiology, Calcium signaling, Neurotoxicity, Cancer

## Abstract

The physical and functional interaction between transient receptor potential channel ankyrin 1 (TRPA1) and neuronal calcium sensor 1 (NCS-1) was assessed. NCS-1 is a calcium (Ca^2+^) sensor found in many tissues, primarily neurons, and TRPA1 is a Ca^2+^ channel involved not only in thermal and pain sensation but also in conditions such as cancer and chemotherapy-induced peripheral neuropathy, in which NCS-1 is also a regulatory component.

We explored the interactions between these two proteins by employing western blot, qRT–PCR, co-immunoprecipitation, Ca^2+^ transient monitoring with Fura-2 spectrophotometry, and electrophysiology assays in breast cancer cells (MDA-MB-231) with different levels of NCS-1 expression and neuroblastoma cells (SH-SY5Y).

Our findings showed that the expression of TRPA1 was directly correlated with NCS-1 levels at both the protein and mRNA levels. Additionally, we found a physical and functional association between these two proteins. Physically, the NCS-1 and TRPA1 co-immunoprecipitate. Functionally, NCS-1 enhanced TRPA1-dependent Ca^2+^ influx, current density, open probability, and conductance, where the functional effects depended on PI3K. Conclusion: NCS-1 appears to act not only as a Ca^2+^ sensor but also modulates TRPA1 protein expression and channel function in a direct fashion through the PI3K pathway. These results contribute to understanding how Ca^2+^ homeostasis is regulated and provides a mechanism underlying conditions where Ca^2+^ dynamics are compromised, including breast cancer. With a cellular pathway identified, targeted treatments can be developed for breast cancer and neuropathy, among other related diseases.

**Supplementary Information:**

The online version contains supplementary material available at 10.1007/s13105-024-01016-z.

## Introduction

In the development from unicellular living forms to complex organisms, the ion calcium (Ca^2+^) stands out as a crucial second messenger that plays a pivotal role in many physiological functions [13]. It controls the cell cycle (death and survival), gene expression, membrane transport, and neurotransmitter release [7]. Ca^2+^-binding proteins, channels, and transporters must be synchronized to transcribe these signals into specific pathways and to achieve homeostasis. Neuronal calcium sensor 1 (NCS-1) is a Ca^2+^-binding protein that was first named as frequenin [47] in the *Drosophila spp.* nervous system. In humans, NCS-1 is mainly found in neurons, especially in the cerebral cortex, the hippocampus [42], and dorsal root ganglion cells [51]. However, this protein is also found in epithelia, adipocytes, neuroendocrine cells, and cardiac myocytes [35, 42, 48].

Four EF-hand motifs with high affinity for Ca^2+^ integrate the molecular structure of NCS-1, which provides it with the ability to detect small changes in intracellular Ca^2+^ concentration and allows it to act as an accurate Ca^2+^ sensor as well as a Ca^2+^-binding protein. Under basal conditions, NCS-1 is bound to Mg^2+^, adopting a spatial configuration that reduces its hydrophobic region exposure and prevents nonspecific reactions [12]. When Ca^2+^is available, it leads to a conformational shift that allows the interaction with an array of binding targets, translating intracellular Ca^2+^concentration changes into biochemical signals [8].

Over the years, many authors have studied the interaction between NCS-1 and a wide pool of target proteins, highlighting novel biological functions and pathophysiological roles in diseases. For instance, this molecular sensor modulates voltage-gated Ca^2+^channel 2.1 (CaV2.1) through its α1 subunit, inducing short-term synaptic facilitation [68]. Additionally, it interacts with the inositol 1,4,5-trisphosphate receptor (InsP3R), increasing the Ca^2+^ signal [56]. Disturbances in this dynamic appears to be involved in several human diseases, including ataxia, seizures, Alzheimer’s disease, Huntington's disease, and cerebral ischemia [52]. Furthermore, NCS-1 has a role as a survival factor through indirect activation of the PI3K signaling pathway [21], which is altered in some forms of cancer [20]. Experiments with overexpression of this Ca^2+^ sensor in breast cancer cell lines resulted in increased invasion and motility with diminished cell–matrix adhesion [1, 37]. This correlates with in vivo studies, where the expression of NCS-1 is higher in breast cancer cells [5], and the level of increase is significantly correlated with shorter survival rates in breast cancer patients [37, 57].

TRPA1 (transient potential receptor channel ankyrin 1) belongs to the TRP family, a versatile nonselective cation channel superfamily. This protein is mainly expressed in small diameter fibers of sensory ganglia [36] and other nonneuronal tissues, such as endothelial cells [19], enterochromaffin cells [29], airway epithelial cells [9], keratinocytes, melanocytes, and fibroblasts. It has been strongly associated with detecting thermal [26, 61] and chemical nociception [76]. Ca^2+^ plays an important role in the modulation of this channel [46, 74] where the increase in intracellular Ca^2+^ activates and potentiates its response to different agonists [36]. Recent evidence suggests that TRPA1 is implicated in developing chemotherapeutic-induced peripheral neuropathy (CIPN), mediating the mechanical and cold hypersensitivity provoked by platinum-based anticancer drugs [44]. Furthermore, TRPA1 is overexpressed in several cancer types, including breast carcinoma. This channel promotes an oxidative stress defense in response to reactive oxygen species (ROS) and Ca^2+^ influx induced by TRPA1 activation reduces chemosensitivity [64].

In this study, we explored the physical and functional interaction between TRPA1 and NCS-1 and investigated how NCS-1 can modulate the expression and electrophysiological activity of TRPA1. These findings provide new insights into the dynamics of these two molecules, which may contribute to understanding cancer and pathophysiological mechanisms.

## Materials and methods

### Cells

The MDA-MB-231 human breast cancer cell line (RRID:CVCL_0062) was obtained from ATCC (American Type Culture Collection, Manassas, VA). This cell line was maintained at 37 °C with 5% CO_2_ in Dulbecco's modified essential medium (DMEM) supplemented with 10% fetal bovine serum (FBS), 1% glutamine, and 1% penicillin/streptomycin. The cells were stably transfected with a previously modified lentivirus and then replicated to generate knockdown (KD) and overexpression (OE) NCS-1 cell lines. The level of NCS1 expression was corroborated, and all cells were passaged for use no more than three months after being thawed and maintained until the start of the experiments.

The SH-SY5Y human cell line (RRID:CVCL_0019) was obtained from ATCC and maintained at 37 °C with 5% CO_2_ in DMEM supplemented with 10% FBS, 1% penicillin/streptomycin, and 1% essential amino acids until the experiments were performed.

### Cell viability assay

Cell viability was determined by the 4,5-dimethylthiazol-2-yl-2,5-diphenyltetrazolium bromide (MTT) assay. Briefly, SH-SY5Y cells were seeded at a density of 2 × 10^4^ cells/well in 96-well plates and incubated in a 37 °C, 5% CO_2_ incubator. After overnight incubation and subsequent reagent exposure, MTT solution (0.5 mg/ml) was added to each well and incubated for 4 h. After incubation, the MTT solution was removed and 100 µl DMSO was added to dissolve the formazan crystals. Absorbance at 570 nm was measured with a microplate reader (ELX800; BioTek Instruments, Inc., Winooski, VT, USA).

### Chemicals and Reagents

All chemicals and solutions were obtained from Sigma–Aldrich (St. Louis, MO), unless otherwise stated. Paclitaxel (PTX), allyl isothiocyanate (AITC), HC-030, H-89 and wortmannin (wort) were dissolved in dimethyl sulfoxide (DMSO) as stock solutions (0.01–1 M). Chelerythrine was dissolved and stored in sterile water.

### Reverse Transcription-Quantitative Polymerase Chain Reaction (qRT–PCR)

Total RNA was extracted from cultured cells using an RNeasy kit (QIAGEN Science, Hilden, Germany) and treated with 55 U RNase-free DNase (QIAGEN Science, Hilden, Germany) following the manufacturer’s instructions. A Nanodrop 2000 spectrophotometer (Thermo Fisher Scientific Inc., Waltham, MA) was employed to determine the purity of the RNA by the 260 nm/280 nm absorbance ratio. RT–qPCR was developed by a StepOnePlus™ Real-Time PCR System (Applied Biosystems), transforming RNA in DNAc using a TaqMan® RNA-to-CT TM 1-Step Kit (Applied Biosystems) following the manufacturer’s instructions. The genes evaluated were TRPA1 (Hs00175798_m1) and β-actin (Hs01060665_g1), which were used as controls. The following protocol was applied: initial denaturation at -50 °C (30 min) and 95 °C (15 min) and 40 cycles of 95 °C (15 s) and 60 °C (60 s). The results were analyzed employing the 2^−ΔΔCt^ method and reported as relative gene expression normalized to the average cycle threshold for the β-actin gene.

### Western blotting

Cultured MDA-MB-231 cells were lysed with protein lysis buffer MPER (Thermo Fisher Scientific Inc., Waltham, MA) supplemented with a protease inhibitor cocktail (P2714, Sigma Aldrich, 1:100) and centrifuged at 10,000 g for 15 min at 4 °C. Cell lysates were separated by SDS–PAGE, followed by electrophoretic transfer onto PVDF membranes. The membranes were cut into three sections, where each section included the molecular weight of the target protein, and each segment was incubated with the corresponding primary antibody. The primary antibodies used were anti-TRPA1 (Alomone, Jerusalem, Israel, 1:500) (Alomone Labs Cat# ACC-037, RRID:AB_2040232), anti-NCS-1 (FL190, Santa Cruz Biotechnology, Santa Cruz, CA, 1:10,000) (Santa Cruz Biotechnology Cat# sc-13037, RRID:AB_649907), and anti-β-actin (Santa Cruz Biotechnology, Santa Cruz, CA, 1:2000) (Santa Cruz Biotechnology Cat# sc-130301, RRID:AB_2223360). Membranes were incubated with primary antibodies overnight at 4 °C. The bands were visualized by an enhanced chemiluminescence system after incubation with rabbit secondary antibody (1:20,000) (Bio–Rad Cat# 166–22,408 EdU) for TRPA1 and NCS-1 and mouse secondary antibody (1:20,000) (Bio–Rad Cat# 170–6516) for β-actin for 2 h at room temperature.

### Co-Immunoprecipitation assays

MDA-MB-231 and SH-SY5Y cells were lysed with protein lysis buffer MPER (Thermo Fisher Scientific Inc., Waltham, MA) supplemented with a protease inhibitor cocktail (P2714, Sigma) and centrifuged at 10,000 g for 15 min at 4 °C. The supernatant was incubated with anti-NCS-1 antibody (FL190, Santa Cruz Biotechnology, Santa Cruz, CA, 1: 5000) for 1 h at 4 °C and then precipitated employing protein-A magnetic beads (PureProteome, EMD Millipore, Billerica, MA) by incubating for 1 h at 4 °C, following the manufacturer's instructions. Beads were washed and then eluted, and protein levels were quantified using western blotting, employing anti-NCS-1 (FL190, Santa Cruz Biotechnology, Santa Cruz, CA, 1: 10,000) or anti-TRPA1 (Alomone, Jerusalem, Israel, 1:500) antibodies. Co-IP experiments were also conducted in reverse mode, employing anti-TRPA1 (Alomone, Jerusalem, Israel, 1:500) to induce immunoprecipitation and then using anti-NCS-1 and anti-TRPA1 in western blot analysis to assess the interactions between NCS-1 and TRPA1.

### Electrophysiology

Whole-cell or inside-out patch-clamp techniques were used to record membrane currents (voltage clamp) in MDA-MB-231 and SH-SY5Y cells. Cells were placed in a recording chamber attached to an inverted microscope (TE2000U, Nikon, Tokyo, Japan). Patch pipettes (Sutter Instruments, Novato, CA) were pulled to resistances of 5–8 MΩ (P-97, Sutter Instruments, Novato, CA) and then polished. After a seal of resistance greater than 5 GΩ was obtained, recordings of membrane currents were made using the whole cell or the inside-out mode. An Axopatch 200B amplifier with a CV203BU headstage (Molecular Devices, Union City, CA) was used. Voltage clamp signals were generated by a Digidata 1440A interface (Molecular Devices, Union City, CA). Acquisition and analysis of signals were performed using pCLAMP 10.0 (Molecular Devices, Union City, CA). A ramp protocol where the membrane potential stepped from a holding potential of − 40 mV to + 100 mV and then ramped to − 100 mV was employed. Open probability (Po) was calculated as the proportion of the total recording time that an ion channel spends in its open state in comparison to its closed state.

All experiments were performed at 20 °C. The standard external solution, which was superfused at 25 °C, contained (mM): 140 NaCl, 5 KCl, 2 CaCl_2_, 1 MgCl_2_, 15 HEPES, and 10 glucose, with the pH adjusted to 7.4 with NaOH. The standard pipette solution contained (mM): 110 KCl, 20 K-gluconate, 20 NaCl, 0.1 CaCl_2_, 4 MgCl_2_, 10 HEPES, 5 glucose, with pH adjusted to 7.1 with NaOH at 25 °C. In Na^+^-free solutions, Na^+^ was replaced with equimolar concentrations of NMDG. To chelate contaminant traces of Ca^2+^, EGTA (1 mM) was added to the Ca^2+^-free solutions.

### Spectrophotometric measurement of Ca.^2+^ by Fura-2

MDA-MB-231 and SH-SY5Y cells in suspension were loaded with Fura-2 AM (5 µmol/l) through incubation in HEPES buffer solution (HBS) for 30 min at 20 °C and then for 15 min at 37 °C. A spectrophotometer was used to measure fluorescence oscillations for 300 s (FP-6500 spectrophotometer, Jasco, Tokyo, Japan). The temperature was maintained at 37 °C, and magnetic stirring was implemented during the measurements with alternating excitation wavelengths of 340 and 380 nm; the fluorescence emission was detected at 510 nm before each experiment. The 380 nm/340 nm signal was calibrated using the Grynkiewicz et al*.* method [23] before each experiment. Briefly, the fluorescence ratio was measured in HBS lacking Ca^2+^ supplemented with EGTA (1 mM) and in HBS supplemented with ionomycin (300 nM) containing 2 mM Ca^2+^, the Ca^2+^ concentration at which Fura-2 is saturated. Maximal and minimal ratios (Rmax and Rmin) were obtained under these two conditions, and the [Ca^2+^]_i_ values were derived using the following equation:

[Ca ^2+^]_i_ = Kd (R − Rmin/Rmax − R)(Sf2/Sb2),

R is the experimentally measured ratio, Sf2 is the fluorescence measured at 380 nm in Ca^2+^-free conditions, and Sb2 is the fluorescence measured at 380 nm with saturating Ca^2+^ (2 mM).

### Isolation and purification of Ca^2+^-free NCS-1 protein

NCS-1 was produced by overexpression of rat NCS-1 in Stratagene BL21(DE3) Codon Plus RIL competent E. coli cells transformed with a pET21-a + bacterial expression vector subcloned with rat NCS-1 cDNA. The published purification protocol (Zozulya et al., 1995) was slightly modified. Briefly, cells were grown at 37 °C in 2 L baffled flasks with 1 L LB Broth (Miller) plus ampicillin (100 µg/mL) and chloramphenicol (30 µg/mL). At an OD595 nm of 0.5–0.7, overexpression was induced with 1 mM isopropyl-dithiogalactoside (IPTG) and incubated for 3 h. Cells were harvested by centrifugation at 3000 rpm for 3 min at 4 °C and resuspended in 10 mL of 50 mM HEPES, 100 mM KCl, 1 mM tris (2-carboxyethyl)phosphine, TCEP), 1 mM MgCl_2_, and 10 mM CaCl_2_ at pH 7.5. Bacteria expressing recombinant NCS-1 were lysed in a buffer containing lysozyme (Sigma Aldrich, 2 mg/mL) and DNase I (from bovine pancreas, Sigma Aldrich, 2 µL/1 mL of 2 mg/mL stock) and subjected to three freeze–thaw cycles using ethanol and dry ice. The lysate was homogenized by sonication for 2 min on ice using a 50% duty cycle and an output level of 5. The lysate was then clarified by centrifugation at 40,000 × g (20,000 rpm, 1 h, 4 °C) and sonicated again to reduce sample viscosity. The supernatant was then filtered with a 0.22 µm Steriflip filter unit before hydrophobic interaction chromatography (HIC). HIC was performed using a GE Health care HiTrap Phenyl HP 5 mL column equilibrated with 50 mM HEPES, 100 mM KCl, 1 mM TCEP, 1 mM MgCl_2_, and 10 mM CaCl_2_ at pH 7.5. After the application of the lysate three times through the column, the column was washed with ten volumes of the same buffer used to equilibrate the column. The recombinant protein was eluted using 50 mM HEPES, 100 mM KCl, 1 mM TCEP, 1 mM MgCl_2_, and 50 mM EDTA at pH 7.5. The protein was collected in 25 × 1 mL fractions and evaluated for purity by SDS–PAGE and Coomassie staining. Recombinant protein fractions were pooled to be desalted using a Bio–Rad Econo-Pac 10DG column with 50 mM HEPES and 100 mM KCl at pH 7.5 as the exchange buffer. NCS-1 was then dialyzed through a series of buffers in a Pierce Slide-A-Lyzer 7 K MWCO cassette: 1 L 10 mM EDTA at pH 2 for 1.5 h; 1 L Milli-Q water for 1.5 h; 1 L 10 mM HEPES at pH 7.4 for 1.5 h; and finally, 1 L 50 mM HEPES, 100 mM KCl, and 0.5 mM TCEP at pH 7.2 overnight. Dialysis was performed using only plastic containers to prevent Ca^2+^ contamination from glass. The protein was concentrated to the desired concentration using a Millipore Ultracel 3 K Amicon Ultra15 centrifugal filter device.

### Statistical analysis

The results are presented as the mean ± standard deviation (SD), or as the median and maximal and minimal values in figures. *n* represents the number of cells tested in the electrophysiological experiments or the number of different cell batches employed in all other assays. Analysis was performed employing GraphPad 9.0 software. Statistically significant differences were determined using one- or two-way ANOVA according to the number of variables, employing Bonferroni post hoc test, where applicable. A p < 0.05 was considered significant, but the exact value of *p* was reported in each analysis.

## Results

### Cell survival

To assess the effects of the chemicals to be used on cell survival, cell viability assessment of MDA-MB231 CTR cells and SH-SY5Y cells in the presence of AITC (300 µM), HC030031 (50 nM), wortmannin (1 µM), H89 (10 µM) and chelerythrine (10 µM) for 6 and 12 h was performed. Any of the chemicals was able to induce a significant cell death (Supplementary Fig. 1).

### NCS-1 modulates the expression of TRPA1

**Fig. 1:**
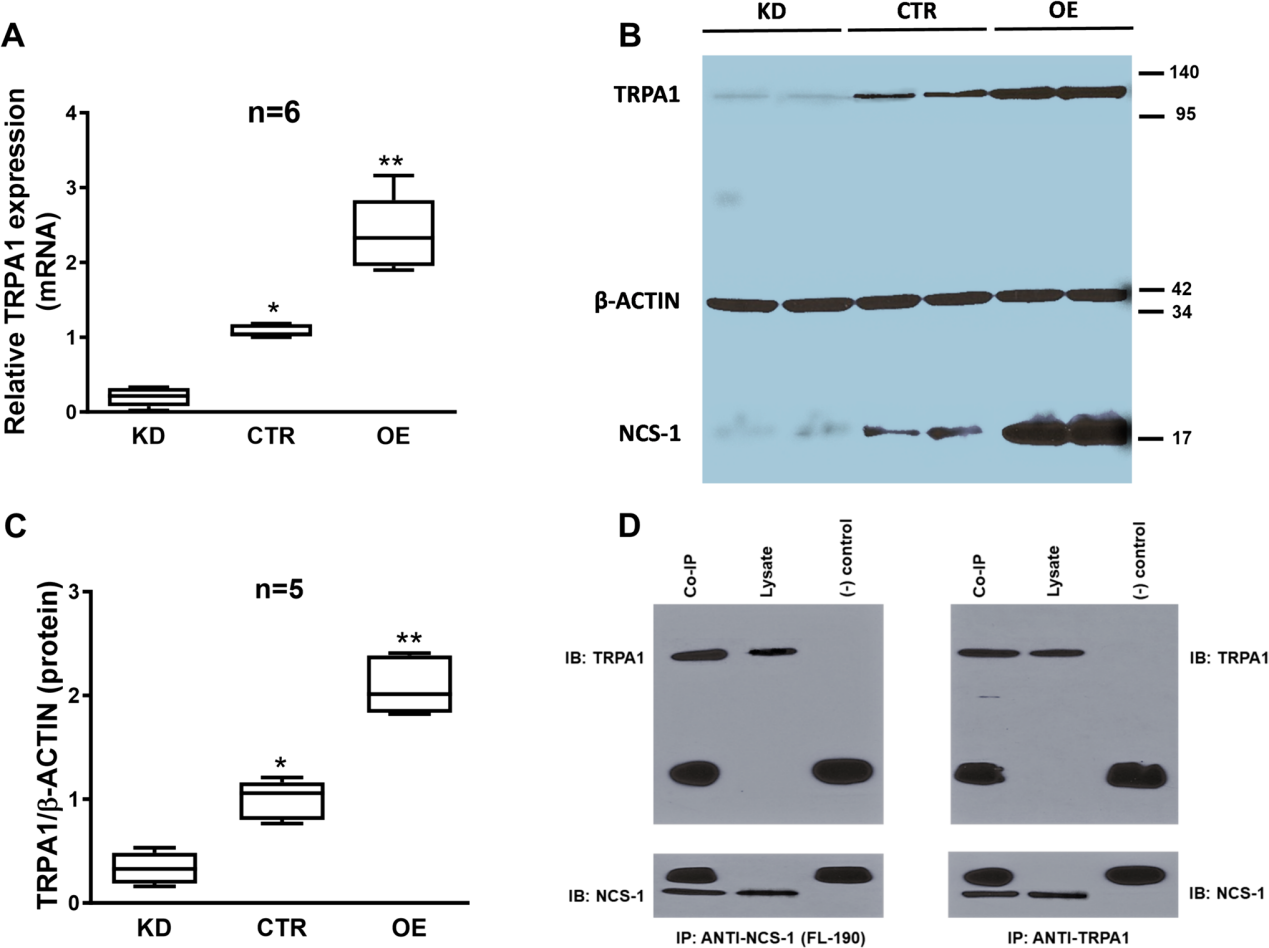
**A**. Comparative expression of TRPA1 mRNA in CTR, KD and OE MDA-MB231, measured by qRT-PCR. Human β-actin was amplified as a control. Note the different levels of expression in the three different MDA-MB231 cell lines. **B**. Representative Western blot showing the expression of TRPA1 protein in the same cell lines in two different batches of cells. Human β-actin was used as a control. Note the different protein levels in the three different MDA-MB231 cell lines. * denotes significance (p < 0.05) comparing with KD cells and ** denotes significance (p < 0.05) comparing with KD and CTR cells. **C**. Comparative levels of TRPA1 expression in MDA-MB231 cells normalized to levels of β-actin expression (n = 5). * denotes significance (p < 0.05) comparing with KD cells and ** denotes significance (p < 0.05) comparing with KD and CTR cells. **D**. Representative Western blot following co-immunoprecipitation as indicated, in CTR MDA-MB231 cells. Note that the non-identified bigger bands correspond to the antibody heavy and light chains in both panels

**Fig. 2:**
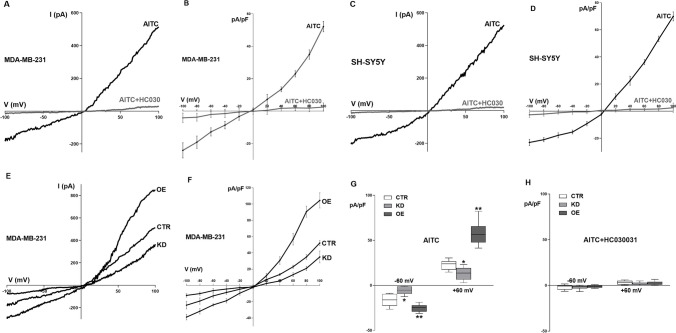
**A**. Typical I-V recording of the current obtained in individual MDA-MB231 CTR cells, elicited by a ramp protocol from -100 mV to + 100 mV and activated by AITC (black trace), which is abolished when the TRPA1 inhibitor HC-030031 is present (gray trace). **B**. I-V relationship of the AITC-induced current in MDA-MB231 CTR cells in the absence (black trace) and presence (gray trace) of HC-030031. **C**. Typical I-V recording of the current obtained in individual SH-SY5Y cells, elicited by a ramp protocol from -100 mV to + 100 mV and activated by AITC (black trace), which is abolished when the TRPA1 inhibitor HC-030031 is present (gray trace). **D**. I-V relationship of the AITC-induced current in SH-SY5Y cells in the absence (black trace) and presence (gray trace) of HC-030031. **E**. Typical I-V recording of the current obtained in individual CTR, KD and OE MDA-MB231 cells, elicited by a ramp protocol from -100 mV to + 100 mV and activated by AITC. **F**. I-V relationship of the AITC-induced current in CTR, KD and OE MDA-MB231 cells. **G**. Comparison between the mean maximal normalized AITC-induced current recorded at -60 mV and + 60 mV in the three different cell lines as indicated in **E** and **F**. **H**. Comparison between the mean maximal normalized AITC-induced current recorded at -60 mV and + 60 mV in the presence of HC-030031 in the three different cell lines. Note that the current was also completely inhibited. n = 12 in all cases. * denotes significant decrease (p = 0.021) and ** denotes significant increase (p = 0.015)

We evaluated the expression of TRPA1 employing qRT–PCR and Western blot in MDA-MB-231 cells with different levels of NCS-1 expression. TRPA1 was increased in cells that overexpressed NCS-1 (OE, mRNA relative expression 2.401 ± 0.443 *p* = 0.001, protein relative expression 2.089 ± 0.25 *p* = 0.003) and lower in cells that underexpressed this protein (KD, mRNA relative expression 0.199 ± 0.112 *p* = 0.0001, protein relative expression 0.334 ± 0.138 *p* = 0.002) in comparison with controls (CTR, mRNA relative expression 1.075 ± 0.074, protein relative expression 0.997 ± 0.169) when measured as either mRNA (Fig. 1A) or protein bands (Fig. 1B and C, Supplemenary Fig. 2A). In Western blot experiments, the expression of TRPA1 was confirmed by identifying a band at 128 kDa, as expected for TRPA1. The expression of β-actin was used as a loading control with a band at 42 kDa. The expression of TRPA1 [53] and NCS-1 [65] in SHSY5Y cells was previously reported.

### NCS-1 immunoprecipitates with the ion channel TRPA1

We explored the molecular interaction between NCS-1 and TRPA1 using co-immunoprecipitation assays in nonmodified MDA-MB-231 cells (CTR). TRPA1 was detected by Western blot in immunoprecipitates pulled down by anti-NCS-1, and NCS-1 was detected by Western blot in immunoprecipitates pulled down by anti-TRPA1, showing the existence of a stable protein–protein interaction (Fig. 1D, Supplementary Fig. 2B, n = 4).

## NCS-1 enhances I_TRPA1_ density, open probability (Po), and conductance (G)

The voltage patch-clamp technique was employed to determine whole-cell currents in MDA-MB-231 CTR and SH-SY5Y cells. The specific TRPA1 agonist AITC (300 µM) elicited a predominantly inward current with a characteristic voltage dependence in both MDA-MB-231 CTR (Fig. 2A and B) and SH-SY5Y cells (Fig. 2C and D). These currents significantly decreased in the presence of the TRPA1 antagonist HC-030031 (HC030, 50 µM), which confirmed its identity as I_TRPA1_.

We used OE, KD, and CTR cells to assess the effects of NCS-1 levels on TRPA1. The currents were higher in OE cells (-25.178 ± 4.088 pA/pF at -60 mV and 57.958 ± 13.051 pA/pF at + 60 mV, p < 0.0001) and lower in KD cells (-5.684 ± 4.511 pA/pF at -60 mV and 13.207 ± 7.076 pA/pF at + 60 mV, p < 0.0001) than in CTR cells (-16.066 ± 6.696 pA/pF at -60 mV and 22.948 ± 5.382 pA/pF at + 60 mV), as shown in a single representative cell (Fig. 2E) or the average of 12 cells (Fig. 2F and G). As with the CTR cell lines, the addition of HC-030031 (50 nM) inhibited the currents with all levels of NCS-1 expression (Fig. 2H, n = 6).

**Fig. 3:**
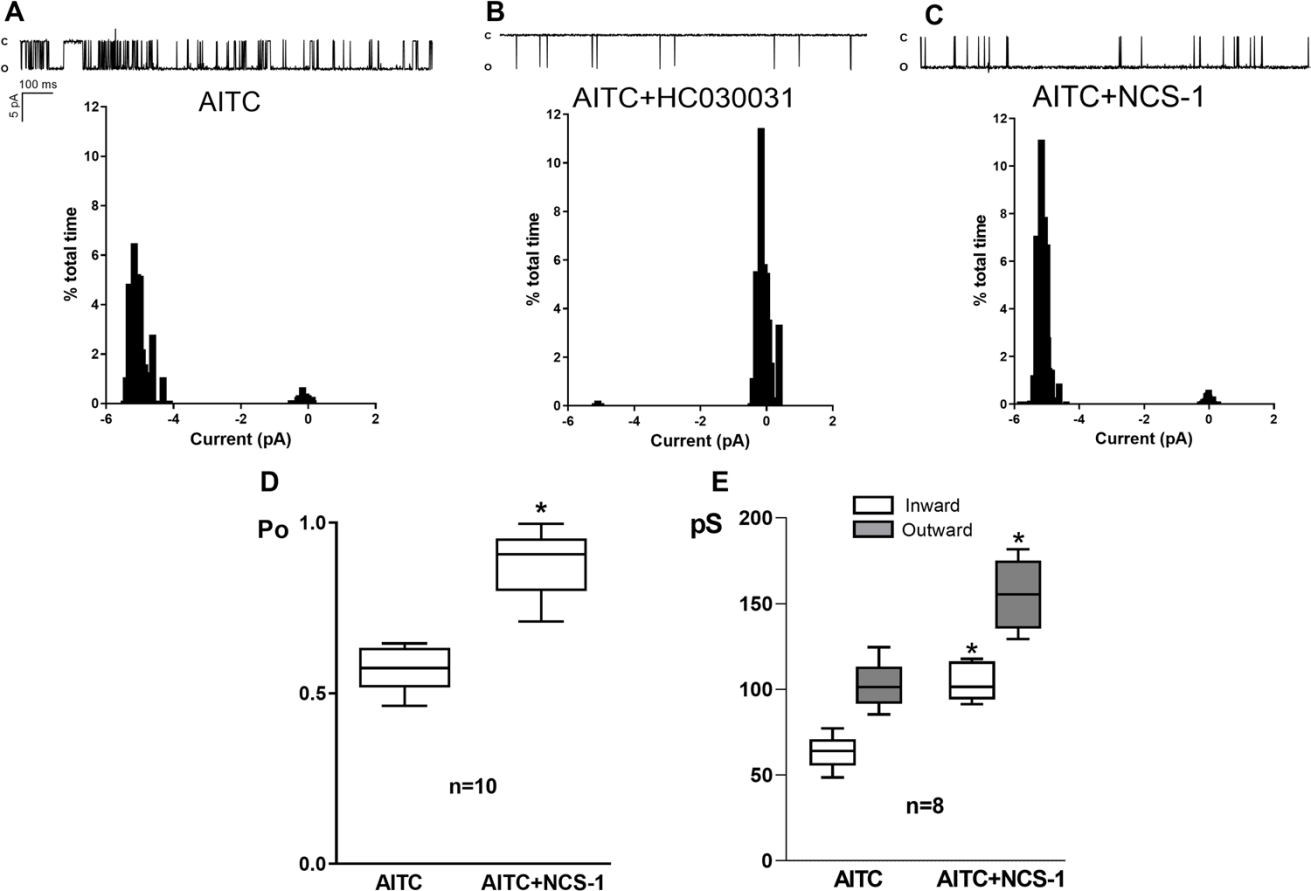
**A**. Recordings of single channel activity (opening is downward) and corresponding amplitude histogram of single-channel currents through TRPA1, activated by AITC, in MDA-MB231 CTR cells, at -60 mV. **B**. Recordings of single channel activity (opening is downward) and corresponding amplitude histogram of single-channel currents through TRPA1, activated by AITC, in the presence of HC030031, at -60 mV. **C**. Recordings of single channel activity (opening is downward) and corresponding amplitude histogram of single-channel currents through TRPA1, activated by AITC, in the presence of NCS1, at -60 mV. **D**. Comparison of the mean open probability (Po) in 10 different patches in the conditions described in A and C. **E**. Comparison of the mean inward (IG_TRPA1_) and outward (OG_TRPA1_) conductances in 8 different patches in the absence and the presence of NCS1, calculated from I-V linear regressions, for each condition. * denotes significance (p = 0.021 for IG_TRPA1_. p = 0.016 for OG_TRPA1_)

Inside-out excised patches were generated from MDA-MB-231 CTR and SH-SY5Y cells to assess the direct effect of NCS-1 on I_TRPA1_. This configuration is necessary because the NCS-1 protein cannot cross the plasma membrane. To determine the NCS-1 concentration to be used, a concentration–response curve was constructed, assessing the effect at -60 mV and + 60 mV, and the minimal concentration that evoked the maximal response was employed (data not shown). NCS-1 (10 μM) was added to the extracellular solution 1 min before recording the currents. AITC (300 µM), alone and in the presence of HC030031 (50 nM) or NCS-1 (10 μM), was added to activate TRPA1 channels and the currents were recorded for 1 min; the corresponding histograms indicating the time that the channel spent in the open and closed states were constructed (Fig. 3A, B and C) and Powas derived in each case. Using MDA-MB-231 CTR cells, TRPA1 Po was significantly increased by NCS-1 (0.883 ± 0.105 vs 0.575 ± 0.07, *p* = 0.0006, Fig. 3D).

**Fig. 4:**
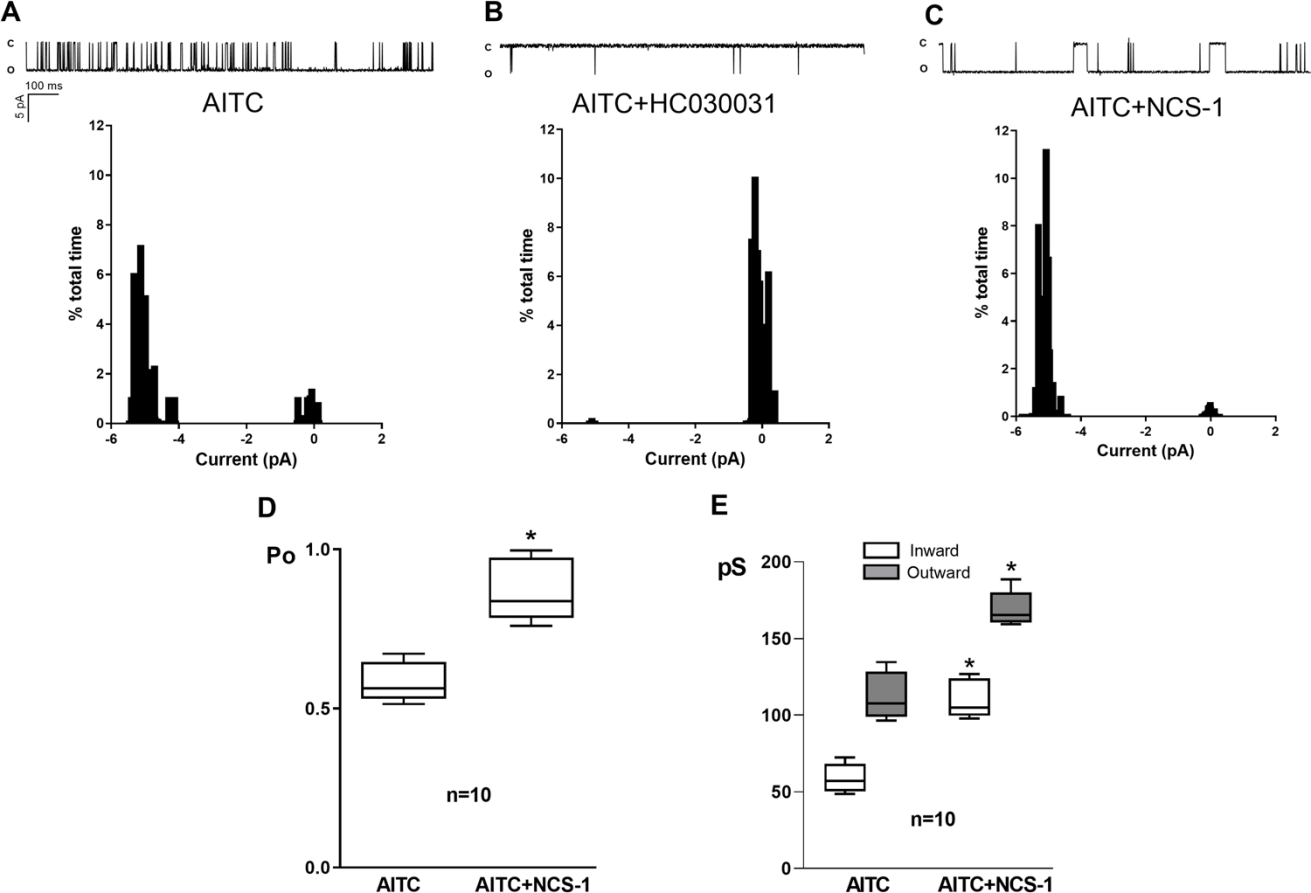
**A**. Recordings of single channel activity (opening is downward) and corresponding amplitude histogram of single-channel currents through TRPA1, activated by AITC, in SH-SY5Y cells, at -60 mV. **B**. **C**. Recordings of single channel activity (opening is downward) and corresponding amplitude histogram of single-channel currents through TRPA1, activated by AITC, in the presence of HC030031, at -60 mV. **C**. Recordings of single channel activity (opening is downward) and corresponding amplitude histogram of single-channel currents through TRPA1, activated by AITC, in the presence of NCS1, at -60 mV. **D**. Comparison of the mean open probability (Po) in 10 different patches in the conditions described in A and C. **E**. Comparison of the mean inward (IG_TRPA1_) and outward (OG_TRPA1_) conductances in 10 different patches in the absence and the presence of NCS1, calculated from I-V linear regressions, for each condition. * denotes significance (p = 0.03 for IG_TRPA1_. p = 0.024 for OG_TRPA1_)

A similar result was obtained with SH-SY5Y cells (Fig. 4). The inward and outward conductances (IG_TRPA1_ and OG_TRPA1_, respectively) were calculated based on the slopes of current–voltage lines; they were consistent with the expected values for TRPA1 currents [46], and both IG_TRPA1_ and OG_TRPA1_ were significantly increased by NCS-1 in both MDA-MB-231 CTR cells (63.424 ± 10.194 pS vs 104.482 ± 11.493 pS for IG_TRPA1_, *p* < 0.0001; 102.254 ± 14.178 pS vs 155.304 ± 20.819 pS for OG_TRPA1_; *p* < 0.0001; Fig. 3E) and SH-SY5Y cells (58.824 ± 9.597 pS vs 110.477 ± 12.817 pS for IG_TRPA1_, *p* < 0.0001; 112.454 ± 15.697 pS vs 169.411 ± 11.755 pS for OG_TRPA1_; *p* < 0.0001; Fig. 4E).

### NCS-1 effects on TRPA1 currents (I_TRPA1_) depend on PI3K pathway activation, but not on PKA or PKC pathways

**Fig. 5:**
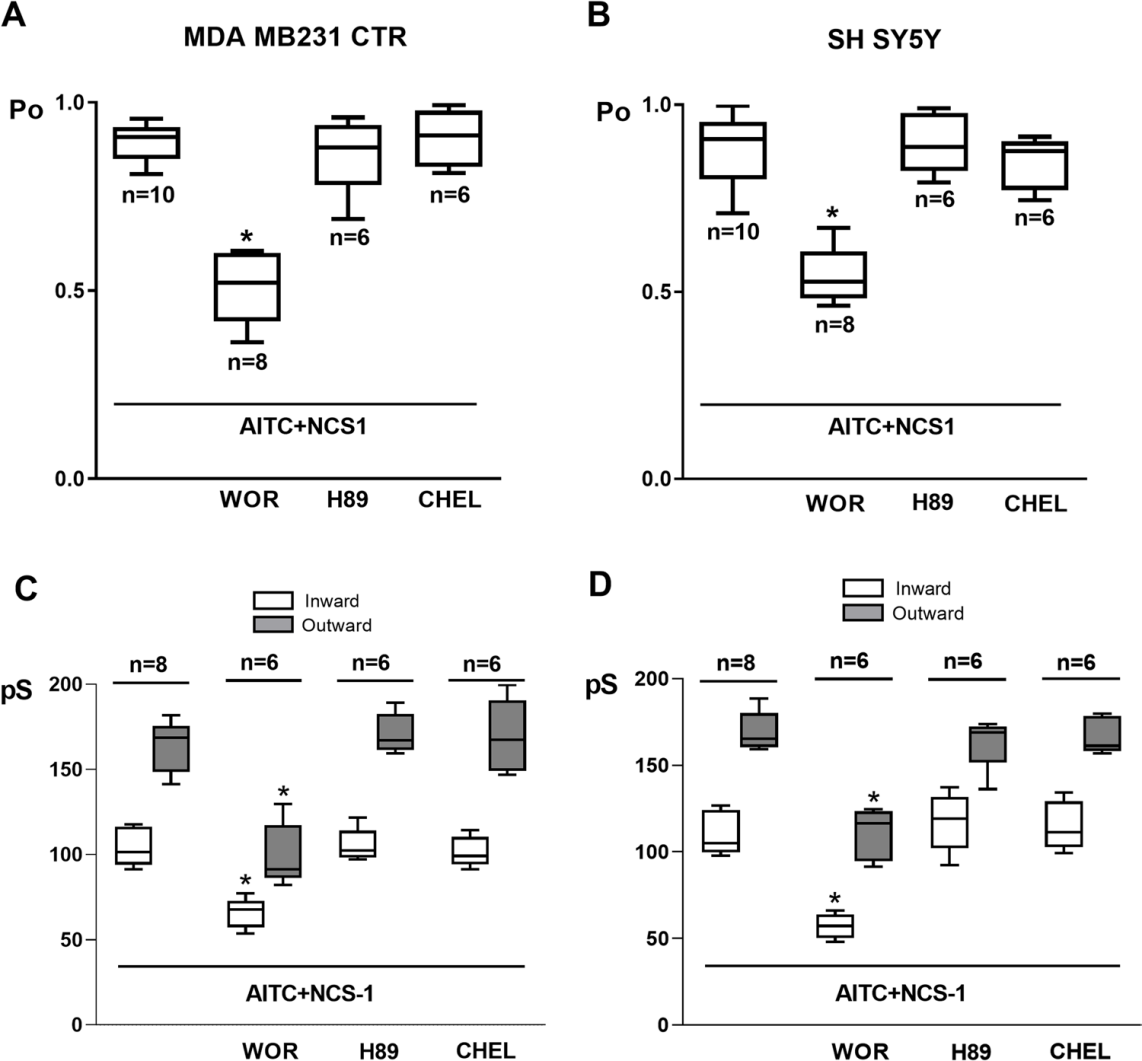
**A**. Comparison of the mean open probability (Po) in 8 different patches from MDA-MB231 CTR cells in the conditions described in A. **B**. Comparison of the mean open probability (Po) in 8 different patches from SH-SY5Y cells in the conditions described in B. **C**. Comparison of the mean inward (IG_TRPA1_) and outward (OG_TRPA1_) conductances in 6 different patches from MDA-MB231 CTR cells in the presence of NCS1 and under the effect of wortmannin (WOR), H89 and chelerythrine (CHEL), calculated from I-V linear regressions. **D**. Comparison of the mean inward (IG_TRPA1_) and outward (OG_TRPA1_) conductances in 6 different patches from SH-SY5Y cells in the presence of NCS1 and under the effect of wortmannin (WOR), H89 and chelerythrine (CHEL), calculated from I-V linear regressions

To evaluate the role of protein kinase A (PKA), protein kinase C (PKC), and phosphoinositide 3-kinase (PI3K), a specific inhibitor of each kinase was added after the addition of NCS-1 to inside-out excised patches. These experiments were performed in patches from MDA-MB-231 CTR and SH-SY5Y cells. The inhibition of PI3K (wortmannin 1 µM, a selective inhibitor at this concentration) attenuated the TRPA1 Po (0.511 ± 0.099 vs 0.895 ± 0.054 in MDA-MB-231 CTR cells, p < 0.0001; 0.547 ± 0.078 vs 0.883 ± 0.105 in MDA-MB-231 CTR cells, p < 0.0001) and the IG_TRPA1_ (104.431 ± 12.817 pS vs 163.304 ± 13.751 pS in MDA-MB-231 CTR cells, p = 0.0005; 110.482 ± 11.493 pS vs 169.101 ± 10.514 pS in SH-SY5Y cells, p < 0.0001) and OG_TRPA1_ (65.624 ± 8.851 pS vs 99.654 ± 16.636 pS in MDA-MB-231 CTR cells, p = 0.0006; 57.024 ± 7.212 pS vs 110.454 ± 13.37 pS in SH-SY5Y cells, p < 0.0001 increase in the presence of NCS-1, observed in the single-cell analysis in both types of cells (Fig. 5A-D), which suggests that this pathway needs to be active to allow NCS-1 effects on I_TRPA1_. In contrast, the inhibition of PKA (H-89 10 µM, a selective PKA inhibitor) and PKC (chelerythrine 10 µM, a selective PKC inhibitor) did not elicit changes in Po (Fig. 5A and C) or G (Fig. 5B and D) in both types of cells.

### NCS-1 increases TRPA1-dependent Ca^2+^ influx and this effect is dependent on PI3K pathway activation, but not on PKA or PKC pathways

**Fig. 6:**
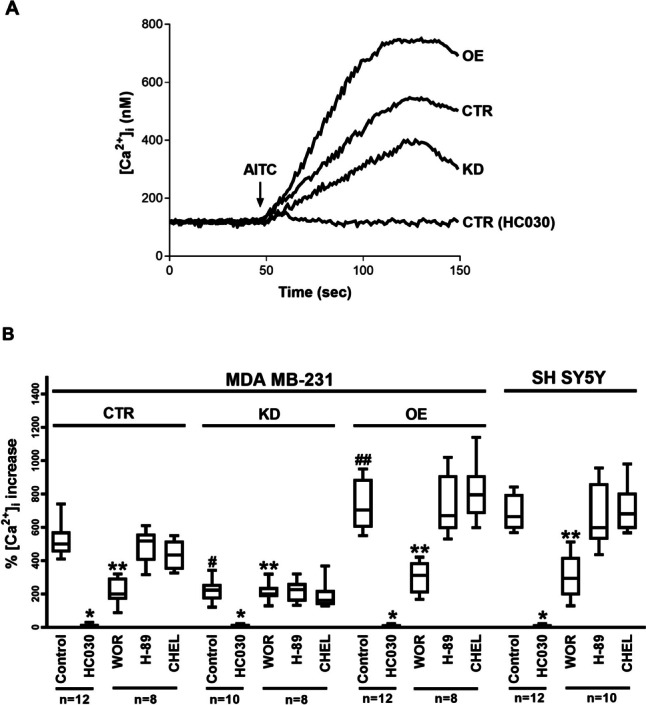
**A**. Representative recordings of intracellular Ca^2+^ concentration in Fura-2-loaded MDA-MB231 (CTR, KD and OE) cells in steady-state conditions and following AITC treatment in the absence and presence of HC030. Fluorescence was recorded for 150 s. The arrow indicates the moment in which AITC was added to the external solution. **B**. Comparison between mean maximal AITC-induced intracellular Ca^2+^ increase percentage in Fura-2-loaded MDA-MB231 cells (CTR, KD, OE) and SH-SY5Y cells, in the absence and presence of HC-030, wortmannin (WOR), H-89 and chelerythrine (CHEL), as indicated. n is indicated in each case. * denotes significant difference with respect to the control situation (following AITC treatment alone) in the same type of cell. ** denotes significant difference with respect to control and HC030-treated cells. ^#^ denotes significant decrease with respect to MDA-MB231 CTR cells. ^##^ denotes significant increase with respect to MDA-MB231 CTR cells

The effects of AITC on intracellular Ca^2+^ concentrations were recorded using suspensions of MDA-MB-231 KD, CTR, and OE cells. This agent significantly increased the intracellular Ca^2+^ concentrations in all three cell lines. However, the increase was higher in OE cells and markedly lower in KD cells than in CTR cells (Fig. 6A and B). AITC also elicited an increase in intracellular Ca^2+^ in SH-SY5Y cells (Fig. 6B). This increase was inhibited by HC030 in all three MDA-MB-231 cell lines and SH-SY5Y cells (Fig. 6A and B), confirming that TRPA1 was the pathway responsible for Ca^2+^. The TRPA1-dependent Ca^2+^ increase in OE and CTR cells and SH-SY5Y cells was lower in the presence of wortmannin but unaffected by treatment with H-89 or chelerythrine. This effect of wortmannin was not observed in KD cells (Fig. 6B).

## Discussion

The present study shows that TRPA1 is expressed in MDA-MB and SH-SY5Y cell lines. Furthermore, our results support the existence of a functional association between this ion channel and NCS-1. We observed a regulatory relationship in which the expression and electrophysiological properties of TRPA1 are regulated by the levels of NCS-1, probably through PI3K pathway activation.

NCS-1 interacts with several proteins and modulates their signaling pathways, executing roles in neurotransmission [28, 50], neuroprotection [40], and nonneuronal processes [41, 72]. However, to date, the TRPA1 channel has not been linked with NCS-1, although some of the most essential functions of TRPA1 appear to require a close relationship with this Ca^2+^ sensor, such as memory [51], neuronal survival [30, 31, 58, 59], cell death [15, 70], Parkinson’s disease [17, 43], CIPN [2, 44], and cancer [18, 55, 63].

There is little knowledge of the mechanisms that lead to transcriptional regulation of TRPA1 [25]. Our results show that the expression of TRPA1 directly correlates with NCS-1 levels. This finding suggests a regulatory role of the NCS-1 protein in the gene expression of the channel. We know that this Ca^2+^ sensor is present in nuclear and perinuclear regions, where it modulates Ca^2+^ levels in cardiomyocytes [69]. Furthermore, NCS-1 regulates gene transcription/stabilization of mRNAs in neurons [33], making this hypothesis reasonable. Nonetheless, NCS-1 might also control gene expression by indirect mechanisms.

TRPA1 is activated by an extensive variety of factors and molecules [36], where many of these factors are irritants [76]. TRPA1 is also strongly regulated by intracellular Ca^2+^ levels, eliciting a bell-shaped response: small and localized increases activate the channel and enhance its response to different agonists [27, 66], whereas higher Ca^2+^ concentrations inactivate it in a process named desensitization or tachyphylaxis [25]. There are several hypotheses by which Ca^2+^ modulates the activity of the TRPA1 channel, either by the presence of direct binding sites in the cytosolic S2-S3 junction [6, 62] or through the intervention of Ca^2+^ sensor proteins such as calmodulin (CaM), where EF3 and EF4 of the C-lobe trigger different degrees of TRPA1 binding and regulation [24]. We hypothesize that NCS-1, as a Ca^2+^ sensor, modulates the activation of the TRPA1 channel in similar ways. First, NCS-1 activates InsP3R, releasing Ca^2+^ from the ER [3, 56, 72], which could lead to direct binding of this ion to TRPA1 and thus increase its activity [10, 16, 75]. Second, given the association between NCS-1 and TRPA1, EF-Hands may exert effects on the channel similar to those described with CaM [24]. Studies with Ca^2+^ and CaM regulating TRPA1 function suggest that the binding location of NCS-1 is probably close to the channel pore, where a slight conformational change in the effector sites could have a significant impact on Po and conductance [39, 67]. Other pathways might also be involved in the activation of the TRPA1 channel downstream of NCS-1 [32].

One of the potential regulatory pathways is the PI3K pathway, where NCS-1 is involved as an upregulating factor. In cortical neurons, NCS-1 mediates cell survival and neurite growth through the activation of PI3K [14, 41, 71]. Another interesting result was obtained in the same type of breast cancer cells used in this study, where NCS-1 elicited enhanced cell survival, motility, and metastatic spread through the PI3K pathway [22, 34]. Here, we demonstrated that NCS-1 activates I_TRA1_ and that this effect is dependent on PI3K activation, highlighting a novel target of the PI3K pathway. Interestingly, PI3K modifies the channel activity of other members of the TRP family, such as TRPV1 in endothelial cells [11] and neurons [60] and TRPC1 in breast cancer cells [73]. Furthermore, the relationship between TRPA1 and PI3K has been shown previously where decreased TRPA1 activity promotes the proliferation and migration of HUVECs, an effect that is abolished by administration of a PI3K inhibitor [32].

MDA-MB-231 cells are a recognized cancer model. In these cells, overexpression of NCS-1 increased migration dynamics, capacity to form distant metastases, and survival in vivo [1], predicting an unfavorable patient outcome [1, 5, 37]. In clinical trials, expression of NCS-1 was also a predictive biomarker for the response to taxane-based neoadjuvant chemotherapy in breast cancer [38], and doxorubicin-induced breast cancer cell death [5]. These NCS-1 dependent effects occur via control of Ca^2+^ signaling, stress, and IP3K pathway activation [21]. It is interesting that TRP channels have also been implicated in similar phenomena, such as the proliferation and migration of breast cancer cells. For example, TRPM7, TRPM8, and TRPV6, have been suggested as biomarkers [49, 54].

NCS1 and TRPA1 have a role not only in cancer but also in the development of neuropathy, a reason to carry out tests in neuronal models such as SH-SY5Y cells. For example, paclitaxel induces the binding of NCS-1 to InsP3R, changing cytosolic Ca^2+^ concentration and triggering intracellular signals that lead to paclitaxel-induced peripheral and central nervous system neuropathy [4, 45].

In summary, our work shows the functional interaction between NCS-1 and TRPA1 in MDA-MB-231 cells, a breast cancer cell line, and SH-SY5Y cells, a neuronal model. We found that the levels of NCS-1 correlate with TRPA1 at both the mRNA and protein levels. Also, the functional properties of TRPA1 are modified by this NCS-1. We also observed that the PI3K pathway, but not PKA or PKC, is involved in this relationship, probably as an effector downstream of NCS-1 activation. These results are important to understanding the mechanisms underlying diverse conditions where Ca^2+^ dynamics are compromised, inspiring further research and developing new treatments for breast cancer and chemotherapy induced neuropathy, among other related diseases.

### Supplementary Information

Below is the link to the electronic supplementary material.

Supplementary file1 Supplementary Figure 1. Cell viability measured with MTT assay in MDA-MB231 CTR cells (A) and SH-SY5Y cells (B) under treatments with AITC (300 µM), HC030031 (HC030, 50 nM), wortmannin (WOR, 1 µM), H89 (10 µM) and chelerythrine (CHEL, 10 µM), for 6 and 12 hours, as indicated. Note that there are no significant differences in any case. (TIF 585 KB)

Supplementary file2 Supplementary Figure 2. A. Uncropped Western blot, from which Figure 1B was extracted. B. Uncropped Western blot, from which Figure 1D was extracted. (TIF 120 KB)

## Data Availability

The datasets generated during and/or analysed during the current study are available from the corresponding author on reasonable request.

## References

[1] Apasu JE, Schuette D, LaRanger R, Steinle JA, Nguyen LD, Grosshans HK, Zhang M, Cai WL, Yan Q, Robert ME (2019). Neuronal calcium sensor 1 (NCS1) promotes motility and metastatic spread of breast cancer cells in vitro and in vivo. FASEB J.

[2] Boeckel GR (1865). Ehrlich BE (2018) NCS-1 is a regulator of calcium signaling in health and disease. Biochimica et Biophysica (BBA)-Acta Molecular Cell Research.

[3] Boehmerle W, Splittgerber U, Lazarus MB, McKenzie KM, Johnston DG, Austin DJ, Ehrlich BE (2006). Paclitaxel induces calcium oscillations via an inositol 1,4,5-trisphosphate receptor and neuronal calcium sensor 1-dependent mechanism. Proc Natl Acad Sci U S A.

[4] Boehmerle W, Splittgerber U, Lazarus MB, McKenzie KM, Johnston DG, Austin DJ, Ehrlich BE (2006). Paclitaxel induces calcium oscillations via an inositol 1, 4, 5-trisphosphate receptor and neuronal calcium sensor 1-dependent mechanism. Proc Natl Acad Sci.

[5] Bong AH, Robitaille M, Milevskiy MJ, Roberts-Thomson SJ, Monteith GR (2020). NCS-1 expression is higher in basal breast cancers and regulates calcium influx and cytotoxic responses to doxorubicin. Mol Oncol.

[6] Brauchi SE, Rothberg BS (2020) Gating and calcium-sensing mechanisms of TRPA1 channels revealed. Cell Calcium 91:102278. 10.1016/j.ceca.2020.102278 10.1016/j.ceca.2020.102278 PMC7530115 32858446

[7] Bultynck G, Parys J (2018). Ca2+ signaling and cell death: Focus on Ca2+-transport systems and their implication in cell death and survival. Cell Calcium.

[8] Burgoyne RD, Haynes LP (2012). Understanding the physiological roles of the neuronal calcium sensor proteins. Mol Brain.

[9] Caceres AI, Brackmann M, Elia MD, Bessac BF, del Camino D, D'Amours M, Witek JS, Fanger CM, Chong JA, Hayward NJ (2009). A sensory neuronal ion channel essential for airway inflammation and hyperreactivity in asthma. Proc Natl Acad Sci.

[10] Cavanaugh E, Simkin D, Kim D (2008). Activation of transient receptor potential A1 channels by mustard oil, tetrahydrocannabinol and Ca2+ reveals different functional channel states. Neuroscience.

[11] Ching L-C, Kou YR, Shyue S-K, Su K-H, Wei J, Cheng L-C, Yu Y-B, Pan C-C, Lee T-S (2011). Molecular mechanisms of activation of endothelial nitric oxide synthase mediated by transient receptor potential vanilloid type 1. Cardiovasc Res.

[12] Choudhary D, Kragelund BB, Heidarsson PO, Cecconi C (2018) The complex conformational dynamics of neuronal calcium sensor-1: a single molecule perspective. Frontiers in molecular neuroscience 11468. 10.3389/fnmol.2018.00468 10.3389/fnmol.2018.00468 PMC6304440 30618617

[13] Clapham DE (2007). Calcium signaling. Cell.

[14] Dason JS, Romero-Pozuelo J, Atwood HL, Ferrús A (2012). Multiple roles for frequenin/NCS-1 in synaptic function and development. Mol Neurobiol.

[15] Deveci HA, Akyuva Y, Nur G, Nazıroğlu M (2019) Alpha lipoic acid attenuates hypoxia-induced apoptosis, inflammation and mitochondrial oxidative stress via inhibition of TRPA1 channel in human glioblastoma cell line. Biomed Pharmacotherapy 111292–304. 10.1016/j.biopha.2018.12.077 10.1016/j.biopha.2018.12.077 30590317

[16] Doerner JF, Gisselmann G, Hatt H, Wetzel CH (2007). Transient receptor potential channel A1 is directly gated by calcium ions. J Biol Chem.

[17] Dragicevic E, Poetschke C, Duda J, Schlaudraff F, Lammel S, Schiemann J, Fauler M, Hetzel A, Watanabe M, Lujan R (2014). Cav1. 3 channels control D2-autoreceptor responses via NCS-1 in substantia nigra dopamine neurons. Brain.

[18] Du G-J, Li J-H, Liu W-J, Liu Y-H, Zhao B, Li H-R, Hou X-D, Li H, Qi X-X, Duan Y-J (2014). The combination of TRPM8 and TRPA1 expression causes an invasive phenotype in lung cancer. Tumor Biology.

[19] Earley S (2012). TRPA1 channels in the vasculature. Br J Pharmacol.

[20] Fresno Vara JA, Casado E, de Castro J, Cejas P, Belda-Iniesta C, Gonzalez-Baron M (2004). PI3K/Akt signalling pathway and cancer. Cancer Treat Rev.

[21] Grosshans HK, Fischer TT, Steinle JA, Brill AL, Ehrlich BE (2020) Neuronal Calcium Sensor 1 is up‐regulated in response to stress to promote cell survival and motility in cancer cells. Mol Oncol 10.1002/1878-0261.12678 10.1002/1878-0261.12678 PMC7266285 32239615

[22] Grosshans HK, Fischer TT, Steinle JA, Brill AL, Ehrlich BE (2020). Neuronal Calcium Sensor 1 is up-regulated in response to stress to promote cell survival and motility in cancer cells. Mol Oncol.

[23] Grynkiewicz G, Poenie M, Tsien RY (1985). A new generation of Ca2+ indicators with greatly improved fluorescence properties. J Biol Chem.

[24] Hasan R, Leeson-Payne AT, Jaggar JH, Zhang X (2017) Calmodulin is responsible for Ca2+-dependent regulation of TRPA1 channels. Sci Rep 7:45098. 10.1038/srep45098 10.1038/srep45098 PMC5362816 28332600

[25] Hatano N, Itoh Y, Suzuki H, Muraki Y, Hayashi H, Onozaki K, Wood IC, Beech DJ, Muraki K (2012). Hypoxia-inducible factor-1α (HIF1α) switches on transient receptor potential ankyrin repeat 1 (TRPA1) gene expression via a hypoxia response element-like motif to modulate cytokine release. J Biol Chem.

[26] Hoffmann T, Kistner K, Miermeister F, Winkelmann R, Wittmann J, Fischer M, Weidner C, Reeh P (2013). TRPA1 and TRPV1 are differentially involved in heat nociception of mice. Eur J Pain.

[27] Jordt S-E, Bautista DM, Chuang H-h, McKemy DD, Zygmunt PM, Högestätt ED, Meng ID, Julius D (2004). Mustard oils and cannabinoids excite sensory nerve fibres through the TRP channel ANKTM1. Nature.

[28] Kabbani N, Negyessy L, Lin R, Goldman-Rakic P, Levenson R (2002). Interaction with neuronal calcium sensor NCS-1 mediates desensitization of the D2 dopamine receptor. J Neurosci.

[29] Kaji I, Yasuoka Y, Karaki S-i, Kuwahara A (2012). Activation of TRPA1 by luminal stimuli induces EP4-mediated anion secretion in human and rat colon. Am J Physiol-Gastrointestinal Liver Physiol.

[30] Koch M, Kreutz S, Böttger C, Grabiec U, Ghadban C, Korf HW, Dehghani F (2011). The cannabinoid WIN 55,212-2-mediated protection of dentate gyrus granule cells is driven by CB1 receptors and modulated by TRPA1 and Cav2. 2 channels. Hippocampus.

[31] Lee SM, Cho YS, Kim TH, Jin MU, Ahn DK, Noguchi K, Bae YC (2012). An ultrastructural evidence for the expression of transient receptor potential ankyrin 1 (TRPA1) in astrocytes in the rat trigeminal caudal nucleus. J Chem Neuroanat.

[32] Li R, Liu R, Yan F, Zhuang X, Shi H, Gao X (2020). Inhibition of TRPA1 promotes cardiac repair in mice after myocardial infarction. J Cardiovasc Pharmacol.

[33] Liss B, Simons C, Benkert J, Deuter N, Pongs O, Schneider T, Duda J (2019) NCS-1 deficiency affects mRNA-levels of genes involved in regulation of ATP-Synthesis and mitochondrial stress in highly vulnerable Substantia nigra dopaminergic neurons. Front Mol Neurosci 12:252. 10.3389/fnmol.2019.00252 10.3389/fnmol.2019.00252 PMC6890851 31827421

[34] Martínez-Rojas VA, Salinas-Abarca AB, Gómez-Víquez NL, Granados-Soto V, Mercado F, Murbartián J (2021) Interaction of NHE1 and TRPA1 Activity in DRG Neurons Isolated from Adult Rats and its Role in Inflammatory Nociception. Neuroscience 465154–165. 10.1016/j.neuroscience.2021.04.025 10.1016/j.neuroscience.2021.04.025 33957206

[35] McFerran BW, Graham ME, Burgoyne RD (1998). Neuronal Ca2+ sensor 1, the mammalian homologue of frequenin, is expressed in chromaffin and PC12 cells and regulates neurosecretion from dense-core granules. J Biol Chem.

[36] Meents JE, Ciotu CI, Fischer MJ (2019). TRPA1: A molecular view. J Neurophysiol.

[37] Moore LM, England A, Ehrlich BE, Rimm DL (2017). Calcium sensor, NCS-1, promotes tumor aggressiveness and predicts patient survival. Mol Cancer Res.

[38] Moore LM, Wilkinson R, Altan M, Toki M, Carvajal-Hausdorf DE, McGuire J, Ehrlich BE, Rimm DL (2018). An assessment of neuronal calcium sensor-1 and response to neoadjuvant chemotherapy in breast cancer patients. NPJ Breast Cancer.

[39] Nagata K, Duggan A, Kumar G, García-Añoveros J (2005). Nociceptor and hair cell transducer properties of TRPA1, a channel for pain and hearing. The J Neurosci: Official J Soc Neurosci.

[40] Nakamura TY, Jeromin A, Smith G, Kurushima H, Koga H, Nakabeppu Y, Wakabayashi S, Nabekura J (2006). Novel role of neuronal Ca2+ sensor-1 as a survival factor up-regulated in injured neurons. J Cell Biol.

[41] Nakamura TY, Nakao S, Wakabayashi S (2016) Neuronal Ca2+ sensor-1 contributes to stress tolerance in cardiomyocytes via activation of mitochondrial detoxification pathways. J Mol Cell Cardiol 99:23–34. 10.1016/j.yjmcc.2016.08.013 10.1016/j.yjmcc.2016.08.013 27555477

[42] Nakamura TY, Wakabayashi S (2012). Role of neuronal calcium sensor-1 in the cardiovascular system. Trends Cardiovasc Med.

[43] Nam JH, Park ES, Won S-Y, Lee YA, Kim KI, Jeong JY, Baek JY, Cho EJ, Jin M, Chung YC (2015). TRPV1 on astrocytes rescues nigral dopamine neurons in Parkinson’s disease via CNTF. Brain.

[44] Nassini R, Gees M, Harrison S, De Siena G, Materazzi S, Moretto N, Failli P, Preti D, Marchetti N, Cavazzini A (2011). Oxaliplatin elicits mechanical and cold allodynia in rodents via TRPA1 receptor stimulation. PAIN.

[45] Nguyen LD, Fischer TT, Ehrlich BE (2021). Pharmacological rescue of cognitive function in a mouse model of chemobrain. Mol Neurodegener.

[46] Nilius B, Prenen J, Owsianik G (2011). Irritating channels: the case of TRPA1. J Physiol.

[47] Pongs O, Lindemeier J, Zhu X, Theil T, Engelkamp D, Krah-Jentgens I, Koch K, Schwemer J, Rivosecchi R, Mallart A (1993). Frequenin—a novel calcium-binding protein that modulates synaptic efficacy in the Drosophila nervous system. Neuron.

[48] Pongs O, Ratai O, Hermainski J, Ravichandran K (2019) NCS-1 deficiency is associated with obesity and diabetes type 2 in mice. Front Mol Neurosci 12:78. 10.3389/fnmol.2019.00078 10.3389/fnmol.2019.00078 PMC6456702 31001084

[49] Prevarskaya N, Zhang L, Barritt G (2007). TRP channels in cancer. Biochimica et Biophysica Acta (BBA)-Mol Basis Dis.

[50] Romero-Pozuelo J, Dason JS, Mansilla A, Baños-Mateos S, Sardina JL, Chaves-Sanjuán A, Jurado-Gómez J, Santana E, Atwood HL, Hernández-Hernández Á (2014). The guanine-exchange factor Ric8a binds to the Ca2+ sensor NCS-1 to regulate synapse number and neurotransmitter release. J Cell Sci.

[51] Saab BJ, Georgiou J, Nath A, Lee FJ, Wang M, Michalon A, Liu F, Mansuy IM, Roder JC (2009). NCS-1 in the dentate gyrus promotes exploration, synaptic plasticity, and rapid acquisition of spatial memory. Neuron.

[52] Sánchez JC, García AM (2018). NCS-1 en la función neuronal. Revista Cubana de Investigaciones Biomédicas.

[53] Sanchez JC, Munoz LV, Galindo-Marquez ML, Valencia-Vasquez A, Garcia AM (2023). Paclitaxel Regulates TRPA1 Function and Expression Through PKA and PKC. Neurochem Res.

[54] Santoni G, Farfariello V (2011). TRP channels and cancer: new targets for diagnosis and chemotherapy. Endocrine, Metabol Immune Disorders-Drug Targets (Formerly Current Drug Targets-Immune, Endocrine & Metabolic Disorders).

[55] Schaefer EA, Stohr S, Meister M, Aigner A, Gudermann T, Buech TR (2013). Stimulation of the chemosensory TRPA1 cation channel by volatile toxic substances promotes cell survival of small cell lung cancer cells. Biochem Pharmacol.

[56] Schlecker C, Boehmerle W, Jeromin A, DeGray B, Varshney A, Sharma Y, Szigeti-Buck K, Ehrlich BE (2006). Neuronal calcium sensor-1 enhancement of InsP 3 receptor activity is inhibited by therapeutic levels of lithium. J Clin Investig.

[57] Schuette D, Moore LM, Robert ME, Taddei TH, Ehrlich BE (2018). Hepatocellular Carcinoma Outcome Is Predicted by Expression of Neuronal Calcium Sensor 1. Cancer Epidemiol Biomarkers Prev.

[58] Shigetomi E, Jackson-Weaver O, Huckstepp RT, O'Dell TJ, Khakh BS (2013). TRPA1 channels are regulators of astrocyte basal calcium levels and long-term potentiation via constitutive D-serine release. J Neurosci.

[59] Shigetomi E, Tong X, Kwan KY, Corey DP, Khakh BS (2012). TRPA1 channels regulate astrocyte resting calcium and inhibitory synapse efficacy through GAT-3. Nat Neurosci.

[60] Stein AT, Ufret-Vincenty CA, Hua L, Santana LF, Gordon SE (2006). Phosphoinositide 3-kinase binds to TRPV1 and mediates NGF-stimulated TRPV1 trafficking to the plasma membrane. J Gen Physiol.

[61] Story GM, Peier AM, Reeve AJ, Eid SR, Mosbacher J, Hricik TR, Earley TJ, Hergarden AC, Andersson DA, Hwang SW, McIntyre P, Jegla T, Bevan S, Patapoutian A (2003). ANKTM1, a TRP-like Channel Expressed in Nociceptive Neurons Is Activated by Cold Temperatures. Cell.

[62] Sura L, Zíma V, Marsakova L, Hynkova A, Barvík I, Vlachova V (2012). C-terminal acidic cluster is involved in Ca2+-induced regulation of human transient receptor potential ankyrin 1 channel. J Biol Chem.

[63] Takahashi N, Chen H-Y, Harris IS, Stover DG, Selfors LM, Bronson RT, Deraedt T, Cichowski K, Welm AL, Mori Y (2018). Cancer cells co-opt the neuronal redox-sensing channel TRPA1 to promote oxidative-stress tolerance. Cancer Cell.

[64] Takahashi N, Chen HY, Harris IS, Stover DG, Selfors LM, Bronson RT, Deraedt T, Cichowski K, Welm AL, Mori Y, Mills GB, Brugge JS (2018). Cancer Cells Co-opt the Neuronal Redox-Sensing Channel TRPA1 to Promote Oxidative-Stress Tolerance. Cancer Cell.

[65] Wang B, Boeckel GR, Huynh L, Nguyen L, Cao W, De La Cruz EM, Kaftan EJ, Ehrlich BE (2016). Neuronal Calcium Sensor 1 Has Two Variants with Distinct Calcium Binding Characteristics. PLoS ONE.

[66] Wang S, Dai Y, Fukuoka T, Yamanaka H, Kobayashi K, Obata K, Cui X, Tominaga M, Noguchi K (2008). Phospholipase C and protein kinase A mediate bradykinin sensitization of TRPA1: a molecular mechanism of inflammatory pain. Brain.

[67] Wang YY, Chang RB, Waters HN, McKemy DD, Liman ER (2008). The nociceptor ion channel TRPA1 is potentiated and inactivated by permeating calcium ions. J Biol Chem.

[68] Weiss JL, Hui H, Burgoyne RD (2010). Neuronal calcium sensor-1 regulation of calcium channels, secretion, and neuronal outgrowth. Cell Mol Neurobiol.

[69] Xiao X, Wu Z-C, Chou K-C (2011). A multi-label classifier for predicting the subcellular localization of gram-negative bacterial proteins with both single and multiple sites. PLoS ONE.

[70] Yazğan Y, Nazıroğlu M (2017). Ovariectomy-induced mitochondrial oxidative stress, apoptosis, and calcium ion influx through TRPA1, TRPM2, and TRPV1 are prevented by 17β-estradiol, tamoxifen, and raloxifene in the hippocampus and dorsal root ganglion of rats. Mol Neurobiol.

[71] Yip PK, Wong L-F, Sears TA, Yáñez-Muñoz RJ, McMahon SB (2010). Cortical overexpression of neuronal calcium sensor-1 induces functional plasticity in spinal cord following unilateral pyramidal tract injury in rat. PLoS Biol.

[72] Zhang K, Heidrich FM, DeGray B, Boehmerle W, Ehrlich BE (2010). Paclitaxel accelerates spontaneous calcium oscillations in cardiomyocytes by interacting with NCS-1 and the InsP3R. J Mol Cell Cardiol.

[73] Zhang LY, Zhang YQ, Zeng YZ, Zhu JL, Chen H, Wei XL, Liu LJ (2020). TRPC1 inhibits the proliferation and migration of estrogen receptor-positive Breast cancer and gives a better prognosis by inhibiting the PI3K/AKT pathway. Breast Cancer Res Treat.

[74] Zurborg S, Yurgionas B, Jira JA, Caspani O, Heppenstall PA (2007). Direct activation of the ion channel TRPA1 by Ca2+. Nat Neurosci.

[75] Zurborg S, Yurgionas B, Jira JA, Caspani O, Heppenstall PA (2007). Direct activation of the ion channel TRPA1 by Ca 2+. Nat Neurosci.

[76] Zygmunt PM, Högestätt ED (2014) Trpa1, Mammalian transient receptor potential (TRP) cation channels, Springer, pp. 583–630. 10.1007/978-3-642-54215-2_23

