# Supplementary figures and images for: NCS-1 protein regulates TRPA1 channel through the PI3K pathway in breast cancer and neuronal cells
Source: J Physiol Biochem. 2024 Apr 2;80(2):451–63. doi: 10.1007/s13105-024-01016-z (PMC11074019; doi:10.1007/s13105-024-01016-z)

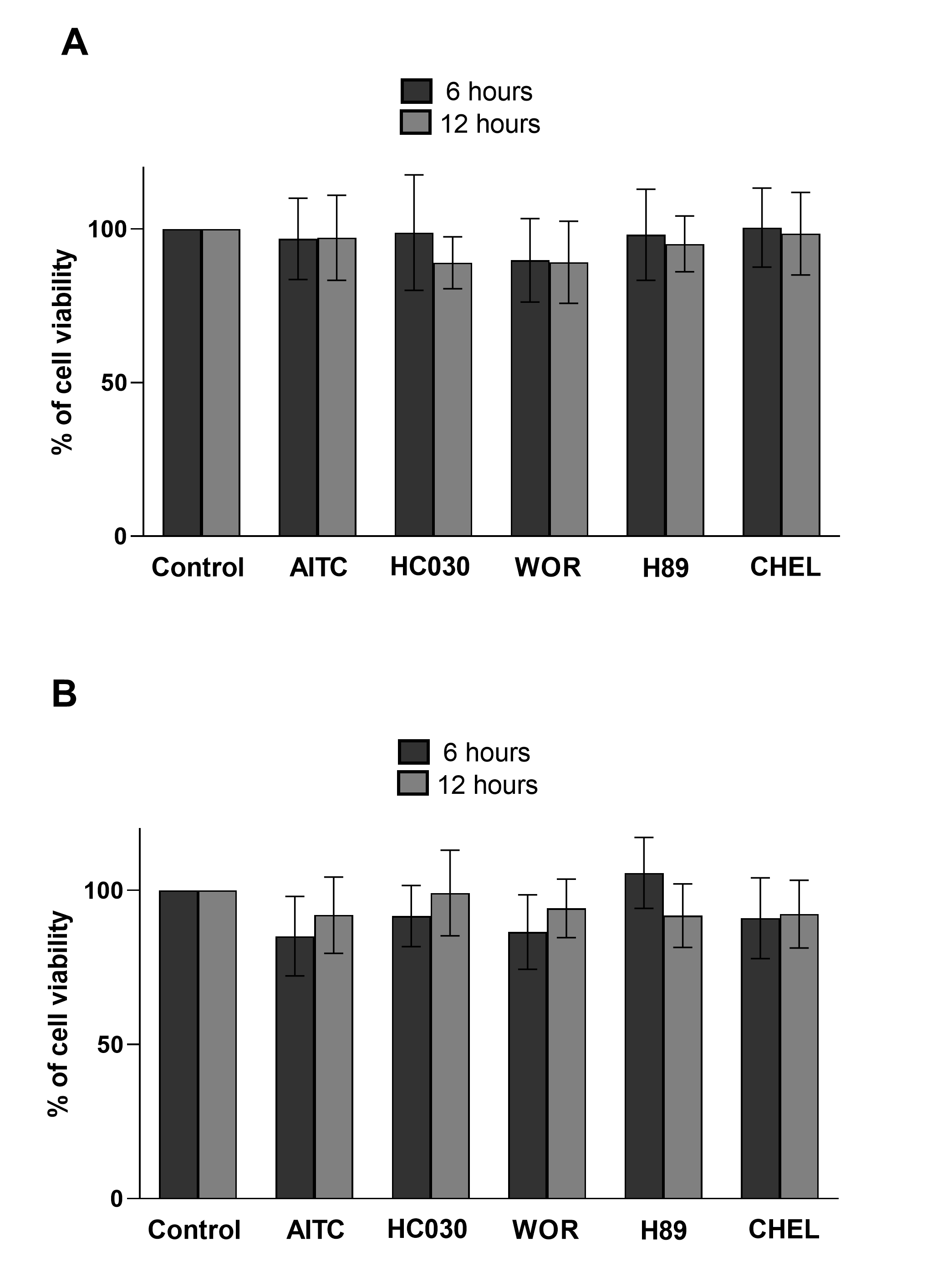

Supplement: Supplementary file 1 — Supplementary file1 Supplementary Figure 1. Cell viability measured with MTT assay in MDA-MB231 CTR cells (A) and SH-SY5Y cells (B) under treatments with AITC (300 µM), HC030031 (HC030, 50 nM), wortmannin (WOR, 1 µM), H89 (10 µM) and chelerythrine (CHEL, 10 µM), for 6 and 12 hours, as indicated. Note that there are no significant differences in any case. (TIF 585 KB) [file 13105_2024_1016_MOESM1_ESM.tif]

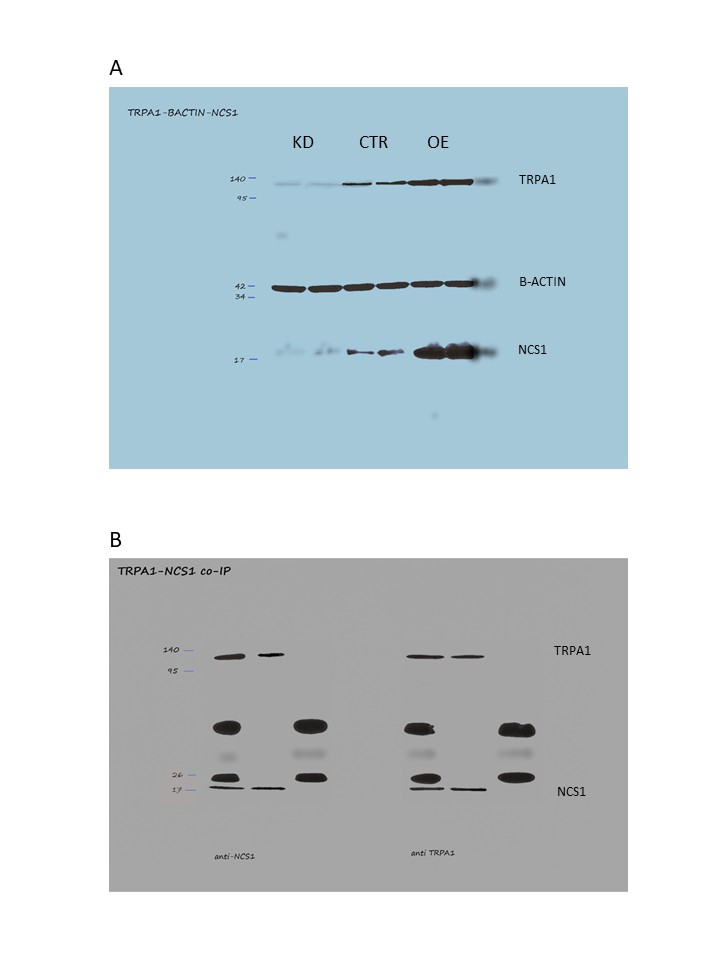

Supplement: Supplementary file 2 — Supplementary file2 Supplementary Figure 2. A. Uncropped Western blot, from which Figure 1B was extracted. B. Uncropped Western blot, from which Figure 1D was extracted. (TIF 120 KB) [file 13105_2024_1016_MOESM2_ESM.tif]
